# The Triple Bottom Line of Sustainable Entrepreneurship and Economic Policy Uncertainty: An Empirical Evidence from 22 Countries

**DOI:** 10.3390/ijerph19137758

**Published:** 2022-06-24

**Authors:** Wentao Gu, Hairui Pan, Zimin Hu, Zhongdi Liu

**Affiliations:** 1 Department of Statistics, Zhejiang Gongshang University, Hangzhou 311121, China; zjgsu-guwentao@hotmail.com (W.G.); csv10165@gmail.com (Z.H.); niipan@163.com (Z.L.); 2 Institute of Quantitative Economics, Zhejiang Gongshang University, Hangzhou 311121, China

**Keywords:** entrepreneurship, sustainability, sustainable entrepreneurship, Economic Policy Uncertainty, Human Development Index

## Abstract

Based on the data of 22 different countries from 2005 to 2018, this paper mainly studies the influence of entrepreneurship on sustainability, and further explores the influence of Economic Policy Uncertainty (EPU) and the interaction effect between EPU and entrepreneurship on sustainability. The results show that EPU can promote economic, environmental, and social development, the interaction between EPU and green entrepreneurship is beneficial to social development, and the interaction between EPU and non-green entrepreneurship inhibits social development. For the heterogeneity analysis, 22 countries are grouped by high and low Human Development Index (HDI). The empirical results find that EPU has a better performance in low HDI countries than that in high HDI countries. In high HDI countries, the interaction between EPU and green entrepreneurship will weaken the positive effects of green entrepreneurship on the environment.

## 1. Introduction

Scholars such as Schumpeter [1] and Knight [2] have put forward the concept of entrepreneurship for a long time, but due to the lack of corresponding research means, entrepreneurship has not been included in economic growth theory. It was not until the 1960s that Arrow [3] and other scholars incorporated technological progress, knowledge spillover, and human capital into the analytical framework of economic growth that entrepreneurship was reflected in the research of economic growth. However, so far, there has not been a generally accepted indicator to measure entrepreneurship [4], so different scholars have different emphases on the specific contents of entrepreneurship. Wong et al. [5] take the number of invention patents divided by GDP as an indicator of innovation activities to measure the innovative spirit of the entrepreneur. He [6] believes that the entry rate and exit rate of enterprises can reflect the intensity of market competition and the difficulty of entering and exiting the market for enterprises. The more active entrepreneurship is, the more frequent enterprises enter and exit. Therefore, in his article, the enterprise entry and exit rate is used to measure entrepreneurship. In the research of Gu, Qian and Lu [7], entrepreneurship is limited to innovation and adventure, which is also a relatively important aspect considered by most mathematicians. There are also many studies that measure several relatively important dimensions of entrepreneurship of individuals or entrepreneurial teams from a psychological perspective in the form of questionnaires and establish an entrepreneurship scale to obtain a more effective measurement [8]. According to Li et al. [9], entrepreneurship is divided into two aspects in this paper, namely, innovative entrepreneurship and business entrepreneurship.

These studies on entrepreneurship, both qualitative and quantitative, only focus on the perspective of economic growth. In recent years, in the face of serious environmental problems such as global warming and ecological environment degradation, people have increasingly realized that it is necessary to make fundamental changes in social production, energy utilization, and the use of natural resources [10]. The role of traditional entrepreneurship in economic transformation is questioned. Some scholars believe that the profit orientation of entrepreneurship leads to environmental degradation [11,12]. However, many researchers do not agree with this view. They believe that although some entrepreneurial activities have some negative effects on society and the environment, they will also play an important role in guiding business activities to realize sustainability [13,14,15]. Tarnanidis et al. [16] studied the contribution of entrepreneurship to preventing environmental pollution. On this basis, Youssefa, Boubaker, and Omri [17] incorporated innovation and government regulation into the Environmental Kuznets Model to retest the contribution of entrepreneurship to preventing environmental pollution. Patzelt and Shepherd [18] believe that entrepreneurial action plays a key role in identifying sustainability opportunities. In addition, Pacheco et al. [19] also believe that entrepreneurs are an important force for social and ecological sustainability. A large number of studies have shown that sustainability is closely related to the sustainability concept held by entrepreneurs. Against this background, sustainable entrepreneurship was proposed. As an emerging field of entrepreneurship research [10,11,20], sustainable entrepreneurship has been heated discussed in recent years. Sustainable entrepreneurship is the extension of sustainability and entrepreneurship. Specifically, it combines the goal of sustainability with traditional entrepreneurship [15]. At present, there is no clear definition of sustainable entrepreneurship. Gast, Gundolf, and Cesinger [21] incorporate entrepreneurship into the model through the extended Environmental Kuznets curve. Kuckertz and Wagner [22] believe that sustainable entrepreneurship needs to keep balance among the triple bottom line, which are economic health, social equity, and environmental resilience. O’Neill et al. [23] believe that sustainable entrepreneurship is a process of business creation, where entrepreneurial activities are connected with social and environmental goals that are related to the realization of sustainable value. Crals and Vereeck [24] defined it as an enterprise’s continuous commitment to ethical behavior, promoting economic development, and improving the quality of life of global residents and future generations.

With regard to the research on sustainable entrepreneurship, many scholars began to pay attention to the triple bottom line from the perspective of sustainability, that is, economic value, ecological (environmental) value, and social value [25,26]. Triple Bottom Line (TBL) is a concept proposed by Elkington in 1994 [27], which aims to measure sustainability. Later, he explained sustainability can be explained mainly by the three values of economic prosperity, environmental quality, and social equity. At present, under the strategy of global sustainability, the concept of TBL has been widely used by many scholars to explain “sustainability”. In fact, the application of TBL is not limited to explaining or describing sustainability in a conceptual way but is also an important tool to measure sustainability [28]. Enterprises play an important role in promoting social goals, improving environmental conditions, and promoting economic development. TBL has been used by researchers in the study of sustainable entrepreneurship [13]. Dhahri and Omri [29] discussed the relationship between entrepreneurship and the long-term and short-term influence of the triple bottom line of sustainability with panel data from 20 countries; Dixon and Clifford [30] developed a model based on society, environment, and economy to test whether enterprises can maintain economic growth without bringing negative effects to the environment and social development. They summarized the balance of society, environment, and economy, which is the main challenge faced by most enterprises. Hall et al. [10] also applied TBL to study entrepreneurs and sustainability. They mentioned that sustainable entrepreneurs should put the three fields of TBL, namely social, environmental, and economic goals, in an equal position. In addition, the value-based model developed by Tilley and Young [15] looks very similar to the TBL model. They believed that sustainable entrepreneurship should be regarded as 12 personal values working together to promote sustainability. Based on the above, this paper uses the triple bottom line to measure sustainability.

It is generally believed that green innovation is closely related to sustainability. Regarding “green innovation”, Driessen and Hillebrand [31] defined it in a quite pragmatic way, which is “do not have to pursue development with the goal of reducing the environmental burden”. Chen et al. [32] defined green innovation as “hardware or software innovation related to green products or processes, including technological innovation related to energy conservation, pollution prevention, waste recycling, green product design or environment management of enterprise”. Asadi et al. [33] demonstrated the importance and potential of green innovation in promoting sustainable performance in the hotel industry. In order to explore the impact of green innovation on sustainability, we divide innovation entrepreneurship into green innovation spirit and non-green innovation spirit, put them into the model for regression, and compare the differences in the results.

Since Baker et al. [34] constructed the economic policy uncertainty index, Economic Policy Uncertainty (EPU) has always been the focus of research. Economic Policy Uncertainty has influences on many aspects, such as the financial market, enterprise investment, and so on. Recent studies have found that EPU also influences entrepreneurship greatly. Nguyen et al. [35] provide strong and consistent evidence that EPU may not always be harmful to entrepreneurship. In the case of uncertainty, the reduction of the number of registered companies does not necessarily mean avoiding starting a business. Tajaddini and Gholipour [36] found that higher levels of EPU are positively associated with higher R&D expenditures per capita as well as innovation outputs (patent applications, patent grants, and trademark applications). He, Ma, and Zhang [37] found that EPU is positively correlated with enterprise innovation on the whole. It is further concluded that in the period with low EPU before 2008, EPU stimulated more innovation activities, but in the period with high EPU after 2008, it inhibited enterprise innovation. Khurana et al. [38] found that higher policy uncertainty is related to the increase in entrepreneurial behavior. Lv [39] provided evidence that policy uncertainty affects entrepreneurs’ special micro individual behavior. Therefore, entrepreneurs are affected by uncertainty in enterprise decision-making all the time. On this basis, it is believed that Economic Policy Uncertainty affects the innovative and entrepreneurial spirit of the entrepreneur. In order to expand the research on economic policy uncertainty (EPU) and entrepreneurship, this paper introduces the Economic Policy Uncertainty index to explore the influence of Economic Policy Uncertainty on entrepreneurship. 

This paper contributes to the literature in the following ways. First, it adds to the current literature on the green innovation spirit. Prior studies have divided entrepreneurship into business entrepreneurship and innovation entrepreneurship [9], and some papers have studied the relationship between green innovation and sustainability [33,40]. On the basis of exploring the impact of entrepreneurship on sustainability, in order to deepen the study of the impact of green innovation on sustainable development, we divide innovation entrepreneurship into green innovation spirit and non-green innovation spirit. We find that in countries with high HDI, a green innovation spirit can significantly reduce environmental pollution, but it was not significant in countries with lower HDI. 

Second, the paper expands research on sustainable entrepreneurship by exploring the impact of EPU on sustainable entrepreneurship. Previous studies have shown that EPU will have an impact on corporate innovation and entrepreneurial activities [37,38]. We add EPU to the regression model and find that EPU can promote the triple bottom line of sustainability, but it weakens the promotion of innovation entrepreneurship to social development. 

The remainder of this paper is structured as follows. Section 2 elaborates on the hypotheses about the impact of entrepreneurship on Sustainability. Section 3 describes the variables selected for this article and the analysis method. Section 4 is the results of regression and Section 5 discusses the empirical results. Section 6 is the conclusion.

## 2. Research Hypotheses

Macroeconomists have long known that modern national economic growth cannot be explained solely by inputs such as capital and labor [41]. Some endogenous growth theorists, such as Romer [42] and Lucas [43], criticized the basic model of the neoclassical production function. They believed that knowledge is the same importance for production as traditional factors such as capital and labor. This has attracted some scholars’ attention to the role of entrepreneurs in identifying and taking advantage of opportunities in a dynamic economy to promote growth [44]. Audretsch and Keilbach [45] introduced Entrepreneurial Capital into the standard Cobb Douglas production function. Through the data of 327 western regions of Germany from 1989 to 1992, it is found that the entrepreneurship of start-ups will bring greater economic growth. Li et al. [9] also introduced entrepreneurship, the variable, into the regression analysis of economic growth, and they also found that entrepreneurship can significantly promote economic growth. In addition, Van Stel and Storey [46] studied 36 developed countries based on a database, and they believed that providing employment through building new companies may serve as the driving factor of entrepreneurship to promote economic growth.

Development economists have always believed that entrepreneurship is crucial to economic growth. However, they focus more on the economic influence of entrepreneurship (GDP, productivity, employment, etc.) while often ignoring its social influence [47]. Therefore, although it is believed that entrepreneurship is the decisive factor in economic growth, it does not directly promote social development. In a large number of economic papers, the influence of entrepreneurship on social development is often ignored [48]. Itri et al. [49] believe that entrepreneurship can improve the quality of social life while reducing costs by creating products and services. York and Venkataraman [50] proposed that entrepreneurship is the way to solve environmental problems rather than the cause of environmental deterioration. Therefore, entrepreneurship is of great significance for the Triple Bottom Line of sustainability. Based on the above analysis, the following hypotheses are proposed.

**Hypothesis** **1** **(H1).** *Entrepreneurship can promote economic development, reduce environmental pollution and improve the quality of social life.*

Many scholars believe that entrepreneurship is more reflected in dealing with uncertainty than risk [2] and that dealing with uncertainty is one of the biggest challenges faced by entrepreneurs. Uncertainty affects all stages of enterprise action, and entrepreneurs’ response to this uncertainty determines the future of enterprises. McMullen and Shepherd [51] believe that judgment under uncertainty is the key factor for the success of entrepreneurial action, which is mainly reflected in two aspects, the ability to understand the uncertainty and the ability to withstand the uncertainty. For uncertainty, entrepreneurs are mainly affected by the uncertainty of national policies [52]. It can be seen that uncertainty has a great influence on entrepreneurship.

The Economic Policy Uncertainty (EPU) index has been a research hotspot since it was put forward. Recent studies have found that EPU also influences entrepreneurship significantly. The research of Nguyen et al. [35] shows that EPU may not always be harmful to entrepreneurship. In the case of uncertainty, the reduction of the number of registered companies does not necessarily mean avoiding starting a business. Other scholars have found that EPU affects the economic behavior of enterprises. These studies found that EPU inhibits enterprise investment significantly [53,54,55]. Therefore, entrepreneurship is under the influence of EPU. Based on the above analysis, the following hypotheses are proposed.

**Hypothesis** **2** **(H2).** *EPU can promote the triple bottom line of sustainability, but the interaction between EPU and entrepreneurship has a negative effect on the triple bottom line of sustainability.*

The Human Development Index (HDI) was first proposed by the United Nations Development Programme (UNDP) in 1990. It is used to measure the level of mankind in several basic aspects, aiming to cover achievements in three basic dimensions, namely life expectancy, education, and living standards. In order to capture these three dimensions, four indicators were determined to measure Human Development Index in 2009. Respectively, life expectancy at birth, adult literacy rate, gross enrollment in primary, secondary and higher education, and per capita GDP adjusted for purchasing power parity.

It is believed in this paper that the influence of entrepreneurship on the Triple Bottom Line of sustainability is different according to regional HDI. Hussain and Dey [56] found that in countries where HDI is less than 0.647, the Human Development Index is positively correlated with carbon dioxide emissions. While in countries where HDI is equal to or greater than 0.647, the Human Development Index is negatively related to carbon dioxide emissions. According to academic papers, developed economies give high priority to applying new technologies, and less developed countries may apply new technologies after a period of time. However, during this period of time, carbon dioxide emissions in low-income and high-income economies may both decline. Therefore, in countries with high HDI and low HDI, there may be heterogeneity in the relationship between the environmental bottom line and the social bottom line of sustainability. Therefore, in countries with different HDI, the impact of EPU on the triple bottom line of sustainability may also be heterogeneous. Based on the above analysis, the following hypotheses are proposed.

**Hypothesis** **3** **(H3).** *Compared with countries with low HDI, EPU’s contribution to the triple bottom line of sustainability is weaker in countries with high HDI.*

## 3. Material and Methods

### 3.1. Variable Selection

#### 3.1.1. Entrepreneurship

According to Li et al. [9], entrepreneurship is limited to two relatively important aspects in this paper, namely Business Entrepreneurship and Innovation Entrepreneurship. These two are important characteristics in entrepreneurship recognized by most scholars.

Business Entrepreneurship (BE) refers to any act of building a new business. At present, it is mainly measured by some indicators such as self-employment ratios, business ownership ratios, business entry, and exit rates, etc. [45,57,58]. This paper takes the entrepreneurial activity (percentage) in the early stage from Global Entrepreneurship Monitor (GEM) as the data for business entrepreneurship.

Innovation Entrepreneurship (IE) is measured primarily by the number of patents or inventions in existing empirical research. For example, Acs [59] used the number of inventions per 1000 people as an indicator of innovative activity, while Wong et al. [5] used the number of invention patents divided by the gross domestic product as an indicator of innovative activity. In general, it is widely used because the number of patents is relatively simple to calculate and easy to obtain. This paper wants to explore the impact of a country’s overall level of innovation on the three pillars, so we used the total number of patents as an explanatory variable rather than a relative index. It is just like how per capita GDP can represent the wealth of a country’s residents. However, it ignores the effects of population, the total GDP can represent the strength of a country. Therefore, the number of national patent applications is used to measure the innovative spirit of entrepreneurs in this paper. 

In order to study the influence of green patents on sustainability, innovation entrepreneurship is further divided into green innovation entrepreneurship and non-green innovation entrepreneurship. Then it compares the differences between them in the influence on sustainability. In this paper, the total number of green patents in various countries represents green innovation entrepreneurship, and non-green innovation entrepreneurship is the difference between the number of green patents and the total number of patents. 

#### 3.1.2. Triple Bottom Line of Sustainability

Economic growth indicators are measured by per capita GDP at purchasing power parity (PPP) published by the World Bank.

Environmental pollution indicators adopt the comprehensive index constructed by the factor analysis method. Referring to Walz [60], three representative greenhouse gas emissions and a characteristic index of the proportion of renewable energy were selected. Therefore, it is composed of four characteristics, namely CO_2_ emissions (metric tons per capita), nitrous oxide emissions (thousand metric tons of CO_2_ equivalent), and methane emissions, and renewable energy consumption (percentage of total final energy consumption). The four characteristics selected in this paper passed the test of KMO and Bartlett before factor analysis. In order to remove the influence of dimension, the characteristic variables were normalized and forward processed. Finally, the comprehensive index constructed represents the degree of environmental pollution. The larger value of the comprehensive index means the higher the degree of pollution.

Social development indicators are also a comprehensive index constructed by factor analysis. Costantini and Monni [61] constructed indicators through the Human Development Index without GDP, which can eliminate the impact of multicollinearity. On this basis, four health characteristics are added. So, it is constructed by seven characteristics, namely life expectancy, average years of education, expected years of education, infant mortality rate (per 1000 live births), incidence of tuberculosis (per 0.1 billion people), risk of impoverishing expenditure for surgical care (percentage of people at risk), and mortality caused by road traffic injury (per 0.1 billion people). The seven characteristics selected for constructing the environmental index also passed the test of KMO and Bartlett before factor analysis, and the characteristic variables were normalized. Finally, the comprehensive index constructed represents the degree of social development. The larger value of the comprehensive index means a higher level of social development.

#### 3.1.3. Economic Policy Uncertainty 

Yusuf [62] affirmed the relationship among uncertainty, entrepreneurial orientation, and performance. House et al. [52] found that entrepreneurs are mainly affected by the uncertainty of the national policy. Yuan et al. [63] studied the influence of EPU on corporate social responsibility participation by taking Chinese listed companies from 2008 to 2015 as a sample. It is found that there is a significant positive correlation between EPU and CSR participation of enterprises. It shows that EPU greatly affects entrepreneurs’ actions, which may affect the Triple Bottom Line of sustainability. Besides, EPU may have an interaction effect, which is also considered in our empirical analysis.

We selected 22 countries in the economic policy uncertainty data jointly released by Stanford University and the University of Chicago from 2005 to 2018 as the research object, namely Australia, Canada, China, Germany, Ireland, Japan, Mexico, Russia, Spain, the United Kingdom, the United States, Brazil, Chile, Colombia, France, Greece, India, Italy, the Netherlands, South Korea, Singapore, and Sweden.

### 3.2. Data Sources of the Samples

The sources of data on Economic Policy Uncertainty include the Economic Policy Uncertainty index jointly published by Stanford University and the University of Chicago, the total early-stage entrepreneurial activity (percentage) published by Global Entrepreneurship Monitor (GEM), the number of green patents in various countries published by WIPO, the Human Development Index from the official website of the United Nations Development Program and others from the World Bank database.

### 3.3. Analysis Method

We test Hypothesis 1, referring to the econometric model setting in the research made by Beck et al. [64], this paper is based on the influence of entrepreneurship on the Triple Bottom Line of sustainability. Besides this, in order to avoid the endogenous impact, this paper takes the explanatory variable as the lag phase for regression according to the actual situation of 22 countries. It makes three regressions on the Triple Bottom Line of sustainability respectively. In order to avoid the influence of dimension, we normalized all variables before regression. The basic panel cointegration model is set as follows.

(1) $$GDP_{i,t}=\alpha_{i}+\beta_{a1}EV_{i,t-1}+\beta_{a2}SV_{i,t-1}+\beta_{a3}IEA_{i,t-1}+\beta_{a4}IEB_{i,t-1}+\beta_{a5}BE_{i,t-1}+e_{i,t}$$

(2) $$EV_{i,t}=\alpha_{i}+\beta_{b1}GDP_{i,t-1}+\beta_{b2}SV_{i,t-1}+\beta_{b3}IEA_{i,t-1}+\beta_{b4}IEB_{i,t-1}+\beta_{b5}BE_{i,t-1}+e_{i,t}$$

(3) $$SV_{i,t}=\alpha_{i}+\beta_{c1}GDP_{i,t-1}+\beta_{c2}EV_{i,t-1}+\beta_{c3}IEA_{i,t-1}+\beta_{c4}IEB_{i,t-1}+\beta_{c5}BE_{i,t-1}+e_{i,t}$$

where *GDP* is the economic bottom line in the triple bottom line, *EV* is the environmental bottom line in the triple bottom line, *SV* is the social bottom line in the triple bottom line, and *IEA* is the economic bottom line in the triple bottom line. This paper divides innovation entrepreneurship into green and non-green parts, where *IEA* is the non-green part and *IEB* is the green part, and BE is business entrepreneurship.

Through the tests in the next chapter, it is sure that these variables are first-order single integers, and there is a long-term panel cointegration relationship between variables. The empirical method brought up by Dhahri and Omri [29] was taken for reference. At this time, OLS cannot be used for estimation, and the result may be biased. Moreover, considering that there may be endogenous problems between variables, Fully Modified Ordinary Least Square (FMOLS) proposed by Pedroni [65] was used to estimate the equation coefficients, so as to obtain consistent parameter estimation. And FMOLS method will be used for all regressions below

Then in order to test Hypothesis 2, we included EPU and its products with Entrepreneurship into the model for regression. In constructing the model, this paper also takes one lag period of explanatory variables for regression to avoid the influence of endogeneity. The basic panel cointegration model set in this paper is as follows. In order to ensure the consistency of the results, we also used FMOLS for regression.

(4) $$\begin{array}{c} GDP_{i,t}=\alpha_{i}+\beta_{d1}EV_{i,t-1}+\beta_{d2}SV_{i,t-1}+\beta_{d3}IEA_{i,t-1}+\beta_{d4}IEB_{i,t-1}+\beta_{d5}BE_{i,t-1}+\beta_{d6}EPU_{i,t-1}\\ +\beta_{d7}EPU*IEA_{i,t-1}+\beta_{d8}EPU*IEB_{i,t-1}+\beta_{d9}EPV*BE_{i,t-1}+e_{i,t} \end{array}$$

(5) $$\begin{array}{c} EV_{i,t}=\alpha_{i}+\beta_{e1}GDP_{i,t-1}+\beta_{e2}SV_{i,t-1}+\beta_{e3}IEA_{i,t-1}+\beta_{e4}IEB_{i,t-1}+\beta_{e5}BE_{i,t-1}+\beta_{e6}EPU_{i,t-1}\\ +\beta_{e7}EPU*IEA_{i,t-1}+\beta_{e8}EPU*IEB_{i,t-1}+\beta_{e9}EPV*BE_{i,t-1}+e_{i,t} \end{array}$$

(6) $$\begin{array}{c} SV_{i,t}=\alpha_{i}+\beta_{f1}GDP_{i,t-1}+\beta_{f2}EV_{i,t-1}+\beta_{f3}IEA_{i,t-1}+\beta_{f4}IEB_{i,t-1}+\beta_{f5}BE_{i,t-1}+\beta_{f6}EPU_{i,t-1}\\ +\beta_{f7}EPU*IEA_{i,t-1}+\beta_{f8}EPU*IEB_{i,t-1}+\beta_{f9}EPV*BE_{i,t-1}+e_{i,t} \end{array}$$

where *EPU* is the economic policy uncertainty, *EPU* ∗ *IEA* is the intersection term of *EPU* and *IEA*, *EPU* ∗ *IEB* is the intersection term of *EPU* and *IEB*, *EPU* ∗ *BE* is the intersection term of EPU and BE. These three items are used to explore the impact of the interaction between EPU and entrepreneurship.

Finally, we test Hypothesis 3. We take the average of the human development index of these 22 countries in the five years from 2014 to 2018, and then take the top 20% countries as the high HDI group and the remaining 80% as the low HDI group. The top four countries were classified into the high HDI group, and the remaining 18 countries were divided into another group. The four countries in the high HDI group are Ireland, Germany, Australia, and Sweden. We used the model above again for regression.

In the next chapter, we will introduce the test of data stationarity before regression and the specific results of the regression.

## 4. Results

### 4.1. Descriptive Statistical Analysis

Table 1 gives the definitional description of each variable and the specific results of the descriptive statistical analysis of the variable, and gives the statistical characteristics of the mean, standard deviation, minimum value, and maximum value, respectively.

It can be seen from the data in Table 1 that the maximum and minimum GDP per capita calculated by purchasing power parity are 10,0581.2 and 2953.11, respectively, indicating that the economic development levels of different countries vary greatly. The standard deviations of non-green entrepreneurial innovation spirit and green entrepreneurial innovation spirit are 57,202.56 and 456.28, respectively, both of which have large standard deviations. This shows that the level of entrepreneurial innovation in different countries is very different. In addition, the uncertainty of economic policy the mean is 128.82, the standard deviation is 66.41, and the maximum and minimum values are 27.00 and 542.76, respectively. Therefore, it can be seen that the Economic Policy Uncertainty varies greatly among different countries and years.

Table 2 shows the correlation coefficients and their significance levels between variables analyzed by Pearson’s test. It can be seen from the table that most of the correlations between the variables studied in this paper are consistent with the actual theory, and are significantly correlated at the 1% level.

According to the judgment method of multicollinearity and the experience of predecessors, it is generally believed that when the correlation coefficient between variables is greater than 0.85, there may be multicollinearity, which will affect the regression results. According to the correlation coefficient between variables shown in the table, the maximum value is 0.7311, and the correlation coefficient of most variables is less than 0.4, much less than 0.85, so it is considered that there is no high degree of multicollinearity. Therefore, according to the correlation analysis, these variables can be used for regression analysis by using the model.

### 4.2. Unit Root Test and Cointegration Test

It can be found that the original variables of these data were not stationary. Therefore, first-order differences were performed on the variables, and then a unit root test was performed on the differenced variables. From the Table 3, it can be found that the test statistic of all variables after differencing becomes significant, so it can be argued that these variables are first-order single integers.

After the unit root test, the cointegration test was continued on the panel data through the two test methods of the Pedroni [65] test and the Kao [66] test. The two most explanatory statistics are Panel ADF-Statistic and Group ADF-Statistic. It can be seen from the Table 4 that the variables basically passed the cointegration test.

### 4.3. Panel Data Cointegration Estimation

To test Hypothesis 1, we will use Equations (1)–(3) for regression. Here are FMOLS estimation results.

As shown in Table 5, Estimating the relationship between variables by FMOLS method, the results for each group are as follows:

In GDP column, it estimates the effects of other variables on economic growth. According to the estimated result of FMOLS, at a 1% level of significance, environmental pollution has a negative impact on GDP (a 1% increase in EV will cause a 0.556% decline in GDP). However, business entrepreneurship is beneficial for GDP growth (a 1% increase in BE will contribute GDP growth by 0.155%). Besides this, social development also contributes to economic growth (a 1% increase in SV will contribute GDP growth by 0.529%). 

In the EV column, it estimates the effects of other variables on environmental pollution. At a 1% level of significance, economic growth will reduce environmental pollution (a 1% increase in GDP will lead to a 0.187% decrease in EV). 

In the SV column, it estimates the effects of other variables on social development. At a 1% level of significance, GDP has a significant impact on social development (1% GDP growth will contribute to SV growth by 0.398%). In addition, the significance of IEA to SV is nearly 5% and non-green innovation can promote social development.

The above results demonstrate the point of view of Hypothesis 1.

### 4.4. Impact of EPU and Entrepreneurship

Then in order to test Hypothesis 2, we included EPU and its products with Entrepreneurship into the model for regression, and we will use Equations (4)–(6) for regression. 

As shown in Table 6, based on the previous study on the interrelationship between entrepreneurship and the triple bottom line of sustainability in international panel data, the influence of EPU on the triple bottom line of sustainability is further examined. Besides this, how the interaction between EPU, entrepreneurial spirit, and innovative spirit influences the triple bottom line of sustainability is explored.

In GDP column, it estimates the effects of other variables on economic growth, and the results show that the environment has a negative influence on GDP growth. Besides this, social development and business entrepreneurship affect economic growth in a positive way, which is consistent with the estimated result of FMOLS above. In addition, after taking EPU into account, it can be seen that EPU has a positive influence on economic growth at the 5% significant level (a 1% increase in EPU will promote a 0.195% increase in GDP). 

In the EV column, it estimates the effects of other variables on the environment. at a 5% level of significance, green innovation entrepreneurship can significantly reduce environmental pollution (a 1% increase in green innovation patents will lead to a 0.225% decrease in EV). In addition, EPU also contributes to reducing gas pollution (a 1% increase in EPU will lead to a 0.111% decrease in EV). 

In the SV column, it estimates the effects of other variables on social development. at a 1% level of significance, EPU has a significantly positive influence on social development (a 1% increase in EPU will promote a 0.225% increase in SV). 

Furthermore, in the SV column, at a 1% level of significance, the interaction between EPU and non-green entrepreneurship will hinder social development. at a 5% level of significance, the interaction between EPU and green entrepreneurship will promote social development.

The above results demonstrate the point of view of Hypothesis 2.

### 4.5. Heterogeneity Analysis

To test Hypothesis 3, the top four countries were classified into the high HDI group, and the remaining 18 countries were divided into another group. We used the model above again for regression.

As shown in Table 7, according to the level of HDI, countries are divided into high HDI countries and low HDI countries for analysis. The above two types of countries will be studied separately through FMOLS analysis. From the perspective of social development, some different results will be discovered between high HDI countries and low HDI countries.

In the SV column, the results show that in countries with high HDI, environmental pollution inhibits social development (a 1% increase in EV will lead to a 0.178% decrease in SV). However, in countries with low HDI, environmental pollution can promote social development (a 1% increase in EV will contribute to a 0.349% increase in SV). 

The above results demonstrate the point of view of Hypothesis 3.

### 4.6. Heterogeneity Analysis of EPU and Entrepreneurship

As shown in Table 8, EPU is considered in heterogeneity analysis. The interaction of EPU and entrepreneurship on the triple bottom line will be different in high HDI countries and low HDI countries.

In the GDP and EV columns, at a 5% level of significance, in countries with high HDI, the interaction between EPU and green innovation will reduce GDP growth, and it will not be beneficial to the environment. In countries with low HDI, the interaction between EPU and business entrepreneurship will hinder economic development.

## 5. Discussion

The experimental results of this paper are shown in Table 5, Table 6, Table 7 and Table 8, the results will be discussed and analyzed in detail.

### 5.1. Panel Data Cointegration Estimation

#### 5.1.1. The Impact of Various Factors on GDP Growth

Environmental pollution has negative effects on GDP growth. As global warming continues to get serious, EV will have a huge impact on climate change and economic development. On the one hand, both gas pollution and climate change have negative impacts on labor productivity, especially in the global agricultural industry. This will force governments and entrepreneurs to take expensive measures to counter negative productivity shocks. On the other hand, governments have to devote a lot of resources to improving the environment in response to climate change and gas pollution.

Business entrepreneurship is beneficial for GDP. Acs [67] elaborated that when entrepreneurs create new businesses, which in turn will create jobs and even increase productivity through technological change. Therefore, high levels of business entrepreneurship will directly translate into high levels of economic growth. Yusuf and Albanawi [68] also pointed out that business entrepreneurship is the engine of economic growth, and it has been regarded as a catalyst for expanding and promoting productive activities in all areas of economic life around the world.

Social development also contributes to GDP growth. Elistia and Syahzuni [69] stated that economic growth and social development are interrelated. On the one hand, economic growth makes it possible to achieve a high level of social development. On the other hand, the improvement of social development levels brings more opportunities for economic growth. In addition, Ranis, Stewart, and Ramirez [70] pointed out that social development is “the goal of social activities and economic growth, and it is also a strategic tool to promote social activities and economic growth”.

#### 5.1.2. Economic Development Helps to Reduce Environmental Pollution

Liobikienė and Butkus [71] conducted an empirical test by extending the EKC model, demonstrating that GDP growth will have a positive impact on the reduction of polluting gas emissions. Generally speaking, a country’s economic growth will initially lead to environmental degradation until a turning point. After that, with the development of new technologies and the government’s support for environmental protection, the environment will be improved.

#### 5.1.3. Economic Growth and Non-Green Innovation Can Promote Social Development

Shome and Tondon [72] investigated the changing relationship between GDP and HDI, and they also examined whether there was a significant correlation between their trends. The results show that higher levels of economic output could directly increase government spending on education, health, and poverty alleviation. That is to say, with the improvement of the economic level, the government’s expenditure on social development and poverty alleviation projects will also increase. Furthermore, de Oliveira Paula and da Silva [73] pointed out that innovation patents can contribute to social development by improving HDI. technological innovation can create better material life for people.

### 5.2. Impact of EPU and Entrepreneurship

#### 5.2.1. The Effects of Other Variables on the Environment

Green innovation entrepreneurship can significantly reduce gas pollution. HN and IM [74] find that when the number of innovations is positive, decreased CO_2_ emissions come from solid fuel consumption and greenhouse gas emissions. This is consistent with the conclusion of this paper that, in the transition to sustainable development, technology innovation is a key driver for reducing environmental pollution.

EPU also contributes to reducing gas pollution. This is because under the circumstance of high policy uncertainty, the government may impose strict policy sanctions and penalties on high-polluting enterprises at any time. Such high pressure will force enterprises to take more measures to reduce pollutant emissions. 

#### 5.2.2. The Impact of EPU and Entrepreneurship on Social Development

EPU will produce a positive influence on social development. In the study by Yang and Chen [75], it was found that EPU has a significantly positive impact on corporate social responsibility (CSR) because investors will be more interested in corporations with high CSR participation. This means that while creating benefits, enterprises will also take responsibility for social development.

The interaction between EPU and non-green entrepreneurship will hinder social development. Research by Panousi and Papanikolaou [76] shows that there is a negative correlation between EPU and innovation. EPU will increase management’s risk aversion, which will make executives become more cautious about innovation decisions to avoid the risk of innovation failure. The research of Lou et al. [77] also shows that EPU will have a negative effect on enterprise innovation. Since corporate inventions bring a series of improvements to the social medical technology and education level, these are all important factors to improve the social development index. So, when EPU interacts with innovative entrepreneurship, EPU can hinder social development by reducing non-green innovation.

The interaction between EPU and green entrepreneurship will promote social development. Tie and Wu [63] stated that EPU will have a positive impact on CSR, that is, during the period of high uncertainty, companies tend to adopt more CSR engagement because it is a positive sign for their stakeholders. Increased investment in CSR will further promote social development. Therefore, when EPU interacts with green entrepreneurship, Enterprises will provide more green products and activities to increase CSR, the interaction can improve the level of social development by improving living condition of residents.

### 5.3. Heterogeneity Analysis

Hussain and Dey [56] pointed out that there is Environmental Kuznets Curve (EKC) relationship between HDI and the environment. When the social development level is within a certain threshold, the SV and environmental pollution are both rising, which represents the positive slope part of the EKC. After the HDI exceeds the threshold level, environmental pollution will decrease when SV rises, which represents the negative slope part of the EKC. 

In high HDI countries, social development is beneficial for the environment. Countries with a high level of social development already exceed the threshold of HDI, the social development will grow slowly after that. The SV is constructed from multiple indicators such as medical health, life expectancy, education level, etc. To continue to grow SV, the environment will be considered an indicator closely related to health and social life, governments and residents will have higher requirements for a high-quality environment and they are willing to pay for a clean environment. Therefore, high HDI countries will give priority to adopting new environmental protection technologies and measures to reduce environmental pollution.

On the contrary, in low HDI countries, social development will be harmful to the environment. Generally speaking, governments are willing to grow GDP at the cost of the environment in these countries, though this way will cause great damage to society in the future. In this way, they can through solve basic social problems to promote social development rapidly. For example, improving the educational level, building more hospitals, preventing infectious diseases, etc. 

### 5.4. Heterogeneity Analysis of EPU and Entrepreneurship

In countries with high HDI, the interaction between EPU and green innovation will hinder GDP growth. entrepreneurs prefer to reform production mode through green innovation so as to supply more high-quality products that meet eco-environmental standards. Compared with low HDI countries, in high HDI countries, green innovation outputs account for a larger share of the economy. However, when EPU occurs, enterprises will be worried about greater losses because of this uncertainty, so they will reduce investment in green innovation. In this case, GDP growth is inhibited.

In low HDI countries, the interaction between EPU and business entrepreneurship will be harmful to GDP growth. Because of inertia and irreversibility, entrepreneurs cannot easily adjust wrong decisions to deal with crises [78]. Businesses will pay the cost of not being able to fully recover and redeploy the investment. EPU will play a negative role in this situation, it will make enterprises reduce investment. Besides this, in countries with low HDI, due to limited resources, the mentality of corporate investors will be more easily affected by EPU. When EPU reduces the business activities of entrepreneurs, social labor employment will also be affected. Therefore, economic development will be influenced.

In countries with high HDI, the interaction between EPU and green innovation will have a negative impact on the environment. Compared with low HDI countries, entrepreneurs usually invest a lot of green innovation in high HDI countries. So, entrepreneurs will be more worried about the adverse influence of EPU, and green innovation will reduce much due to EPU. From this perspective, although green innovation is helpful to reduce environmental pollution, the interaction is not good for the environment in high HDI countries. 

## 6. Conclusions

Based on data from 2005 to 2018, this paper takes 22 international regions with different levels of development as research objects. Then, it explores the internal interaction of the triple bottom line of sustainability, including economic, environmental, and social development. This paper also discusses the influence of Innovation Entrepreneurship and Business Entrepreneurship on sustainability. The paper also explores the interaction of EPU and entrepreneurship effect on the triple bottom line of sustainability. The empirical results are shown as follows:

Through the above empirical analysis results, it can be seen that entrepreneurship will significantly accelerate economic development and social progress. Besides this, green innovation entrepreneurship can significantly reduce environmental pollution. Therefore, entrepreneurship plays a strong role in promoting sustainability.

EPU has a positive impact on the triple bottom line of sustainability. The coordination between EPU and green innovation entrepreneurship promotes social development. In addition, EPU inhibits the positive effect of non-green innovation entrepreneurship on social development. 

By setting the threshold of the social development index and analyzing the heterogeneity, some differences are shown. In countries with high HDI, improving social development levels can reduce environmental pollution and, in turn, the reduction of environmental pollution can promote social development. 

In view of the fact that green sustainable development has become the demand of the new times, this paper puts forward some suggestions for enterprises and governments as follows based on the above conclusion: 

From the perspective of enterprises: Enterprises are advised to regard green development as corporate social responsibility. This action not only helps protect the environment but also creates a good social reputation for enterprises, which enables the enterprises to be favored by investors and get more financing. In addition, enterprises are encouraged to develop material-saving and energy-saving products through technology innovation. This is conducive to reducing cost, enhancing competitiveness, seeking market opportunities, and expanding market share.

From the perspective of governments: For the purpose of building a clean, low-carbon, and environment-friendly social environment, governments should take some measures. Firstly, in order to urge enterprises to give up the traditional production mode, which is high energy consumption and high pollution. governments should improve relevant policies on technological innovation to reduce the burden of enterprises in terms of green technological innovation. Secondly, the government should advocate the economic concept of green and sustainable development, which is not only helpful to environmental protection but also greatly conducive to social and economic development in the long run. In addition, the research in this paper shows that EPU and innovation have significant effects on the triple bottom line of sustainability, so the stability of government policies on encouraging innovation will also promote enterprises to take the green sustainable path.

## Figures and Tables

**Table 1:** Descriptive statistical analysis of variables.

Variable	Variable Interpretation	Mean	sd	min	Max
GDP	per capita GDP at purchasing power parity	34,075.41	17,868.54	2953.11	100,581.2
EV	Environmental pollution variable	0.24	0.19	0	1
SV	Social development variable	0.78	0.18	0	1
IEA	no-green Innovative entrepreneurs: number of non-green patent applications	29,305.04	57,202.56	9.24	311,327.01
IEB	green Innovative entrepreneurs: number of green patent applications	264.19	456.28	0.97	2346.77
BE	Business Entrepreneurship: Total Early-Stage Entrepreneurial Activity	9.67	5.52	2.20	27.40
EPU	Economic policy uncertainty	128.82	66.41	27.00	542.76

**Table 2:** Correlation coefficient table.

Variable	GDP	EV	SV	IEA	IEB	BE	EPU
GDP	1.0000						
EV	0.5544	1.0000					
SV	0.7311	0.3998	1.0000				
IEA	0.0798	0.1315	−0.0581	1.0000			
IEB	0.2261	−0.0087	0.2221	0.7056	1.0000		
BE	−0.3129	−0.0898	−0.3187	0.1344	−0.1834	1.0000	
EPU	0.1723	0.0078	0.1531	0.0544	0.1121	−0.0004	1.0000

**Table 3:** Table Unit root test of variables.

Method	HT Test	Breitung Test	IPS Test
dGDP	0.127 *** (0.000)	−6.277 *** (0.000)	−5.585 *** (0.000)
dEV	−0.241 *** (0.000)	−8.283 *** (0.000)	−6.952 *** (0.000)
dSV	−0.199 *** (0.000)	−7.468 *** (0.000)	−6.695 *** (0.000)
dIEA	0.042 *** (0.000)	−5.714 *** (0.000)	−6.062 *** (0.000)
dIEB	0.393 *** (0.000)	−7.537 *** (0.000)	−3.342 *** (0.000)
dBE	−0.434 *** (0.000)	−8.024 *** (0.000)	−9.045 *** (0.000)

Note. *** Represents significance at the 1 percent level.

**Table 4:** Panel cointegration test.

Method	Panel ADF-Statistic	Group ADF-Statistic	Kao-Statistic
GDP	0.429 ** (0.012)	−2.246 ** (0.012)	0.848 (0.198)
EV	−5.467 *** (0.000)	−4.761 *** (0.000)	−0.057 (0.477)
SV	−1.147 *** (0.001)	−3.344 *** (0.000)	−0.683 (0.247)
IEA	−0.929 *** (0.001)	−3.566 *** (0.000)	0.002 (0.498)
IEB	−2.928 *** (0.000)	−5.426 *** (0.000)	−5.079 *** (0.001)
BE	−10.391 *** (0.000)	−12.094 *** (0.000)	1.017 (0.154)

Note. ***, ** Represent significance at the 1 percent and 5 percent levels, respectively.

**Table 5:** FMOLS estimation results.

Variable	GDP	EV	SV
GDP		−0.187 *** (0.001)	0.398 *** (0.000)
EV	−0.556 *** (0.000)		0.154 (0.124)
SV	0.529 *** (0.000)	0.065 (0.373)	
IEA	0.080 (0.238)	0.003 (0.923)	0.096 (0.057)
IEB	0.061 (0.671)	−0.067 (0.431)	0.123 (0.258)
BE	0.155 *** (0.000)	−0.001 (0.980)	0.011 (0.710)
ADJ-R^2	0.944	0.975	0.969

Note. *** Represents significance at the 1 percent level.

**Table 6:** Impact of EPU and Entrepreneurship.

Variable	GDP	EV	SV
GDP		−0.120 ** (0.041)	0.289 *** (0.000)
EV	−0.437 *** (0.001)		0.173 (0.063)
SV	0.467 *** (0.000)	0.087 (0.240)	
IEA	0.085 (0.465)	0.050 (0.450)	0.284 *** (0.000)
IEB	0.178 (0.370)	−0.225 ** (0.046)	−0.062 (0.647)
BE	0.211 *** (0.000)	-0.020 (0.578)	0.029 (0.505)
EPU	0.195 ** (0.044)	−0.111 ** (0.044)	0.225 *** (0.000)
EPU∗IEA	−0.117 (0.825)	−0.216 (0.469)	−1.178 *** (0.001)
EPU∗IEB	−0.215 (0.649)	0.416 (0.119)	0.805 ** (0.012)
EPU∗BE	−0.309 (0.153)	0.120 (0.330)	−0.195 (0.193)
ADJ-R^2	0.945	0.975	0.970

Note. ***, ** Represent significance at the 1 percent and 5 percent levels, respectively.

**Table 7:** Heterogeneity analysis.

Variable	GDP		EV		SV	
	HIGH HDI Countries	LOW HDI Countries	HIGH HDI Countries	LOW HDI Countries	HIGH HDI Countries	LOW HDI Countries
GDP			0.160 (0.191)	−0.129 (0.053)	0.158 *** (0.000)	0.553 *** (0.000)
EV	0.317 (0.271)	−0.365 ** (0.016)			−0.178 *** (0.000)	0.349 ** (0.039)
SV	3.162 *** (0.000)	0.396 *** (0.000)	−1.158 *** (0.005)	0.104 (0.083)		
IEA	0.003 (0.991)	0.093 (0.067)	−0.021 (0.879)	−0.009 (0.765)	−0.013 (0.812)	0.084 (0.135)
IEB	1.046 (0.667)	0.127 (0.230)	−2.032 (0.079)	−0.076 (0.264)	−0.153 (0.739)	0.092 (0.434)
BE	−0.006 (0.981)	0.148 *** (0.000)	−0.161 (0.236)	0.004 (0.843)	0.121 ** (0.026)	−0.015 (0.667)
ADJ-R^2	0.608	0.970	0.973	0.977	0.793	0.968

Note. ***, ** Represent significance at the 1 percent and 5 percent levels, respectively.

**Table 8:** Heterogeneity analysis of EPU and Entrepreneurship.

Variable	GDP		EV		SV	
	HIGH HDI Countries	LOW HDI Countries	HIGH HDI Countries	LOW HDI Countries	HIGH HDI Countries	LOW HDI Countries
GDP			0.164 *** (0.158)	−0.002 (0.971)	0.117 *** (0.001)	0.369 *** (0.000)
EV	0.406 (0.124)	−0.156 (0.339)			−0.163 *** (0.000)	0.470 *** (0.004)
SV	2.891 *** (0.000)	0.315 *** (0.000)	−1.053 *** (0.007)	0.141 ** (0.011)		
IEA	−0.739 (0.237)	0.139 (0.107)	0.049 (0.859)	0.013 (0.790)	0.198 ** (0.034)	0.248 *** (0.005)
IEB	5.302 (0.101)	0.188 (0.195)	−4.031 *** (0.009)	−0.228 *** (0.005)	−0.386 (0.443)	−0.001 (0.994)
BE	−0.559 (0.284)	0.209 *** (0.000)	-0.031 (0.892)	-0.019 (0.462)	0.227 *** (0.004)	0.003 (0.943)
EPU	−0.200 (0.766)	0.200 *** (0.006)	−0.129 (0.670)	−0.157 *** (0.000)	0.221 ** (0.030)	0.254 *** (0.000)
EPU∗IEA	2.897 (0.202)	−0.312 (0.453)	−0.449 (0.660)	−0.109 (0.649)	−0.975 *** (0.004)	−1.053 ** (0.013)
EPU∗IEB	−14.660 ** (0.046)	0.004 (0.988)	6.842 ** (0.044)	0.350 (0.085)	1.532 (0.170)	0.603 (0.095)
EPU∗BE	3.465 (0.128)	−0.339 ** (0.028)	−0.277 (0.786)	0.147 (0.104)	−0.557 (0.105)	−0.198 (0.221)
ADJ-R^2	0.606	0.971	0.973	0.980	0.796	0.970

Note. ***, ** Represent significance at the 1 percent and 5 percent levels, respectively.

## Data Availability

The datasets used and analyzed during this study are available from the corresponding author on reasonable request.
